# Expression Profiles of SnoN in Normal and Cancerous Human Tissues Support Its Tumor Suppressor Role in Human Cancer

**DOI:** 10.1371/journal.pone.0055794

**Published:** 2013-02-13

**Authors:** Nadine S. Jahchan, Gaoliang Ouyang, Kunxin Luo

**Affiliations:** 1 Department of Molecular and Cell Biology, University of California, Berkeley, California, United States of America; 2 College of Life Sciences, Xiamen University, Xiamen, Fujian, China; Ottawa Hospital Research Institute, Canada

## Abstract

SnoN is a negative regulator of TGF-β signaling and also an activator of the tumor suppressor p53 in response to cellular stress. Its role in human cancer is complex and controversial with both pro-oncogenic and anti-oncogenic activities reported. To clarify its role in human cancer and provide clinical relevance to its signaling activities, we examined SnoN expression in normal and cancerous human esophageal, ovarian, pancreatic and breast tissues. In normal tissues, SnoN is expressed in both the epithelium and the surrounding stroma at a moderate level and is predominantly cytoplasmic. SnoN levels in all tumor epithelia examined are lower than or similar to that in the matched normal samples, consistent with its anti-tumorigenic activity in epithelial cells. In contrast, SnoN expression in the stroma is highly upregulated in the infiltrating inflammatory cells in high-grade esophageal and ovarian tumor samples, suggesting that SnoN may potentially promote malignant progression through modulating the tumor microenvironment in these tumor types. The overall levels of SnoN expression in these cancer tissues do not correlate with the p53 status. However, in human cancer cell lines with amplification of the *snoN* gene, a strong correlation between increased SnoN copy number and inactivation of p53 was detected, suggesting that the tumor suppressor SnoN-p53 pathway must be inactivated, either through downregulation of SnoN or inactivation of p53, in order to allow cancer cell to proliferate and survive. These data strongly suggest that SnoN can function as a tumor suppressor at early stages of tumorigenesis in human cancer tissues.

## Introduction

SnoN (Ski-novel protein) was fist discovered as an oncoprotein that can induce transformation of chicken and quail embryonic fibroblasts when overexpressed [1]–[4]. In mammals, SnoN contains both pro-oncogenic and anti-oncogenic activities. The pro-oncogenic activity of SnoN is dependent on its ability to bind to the Smad proteins and antagonize the cytostatic responses of TGF-β [5], [6]. Suppporting its pro-oncogenic role, SnoN expression is elevated in most human cancer cell lines, and reduction of SnoN expression by shRNA in invasive breast cancer and lung cancer cell lines inhibits tumor growth both *in vitro* and *in vivo* [7]. More importantly, overexpression of SnoN in the mouse mammary gland accelerates breast cancer development and pulmonary metastasis induced by the Polyoma middle T antigen (PyVmT) [8], providing the first *in vivo* support for the pro-oncogenic activity of SnoN. In recent years, however, more and more evidence have emerged indicating that SnoN can also act as a tumor suppressor. Heterozygous mice lacking one copy of the *snoN* gene show an increased susceptibility to carcinogen-induced tumorigenesis, suggesting that SnoN may protect mice from carcinogenesis [9]. We have recently shown that high levels of SnoN inhibit oncogene-induced transformation of primary mouse embryonic fibroblasts *in vitro* and block tumor development *in vivo* in a two-step skin carcinogenesis mouse model [10]. This anti-oncogenic activity of SnoN is independent of its ability to repress TGF-β signaling and likely stems from its ability to induce stabilization of p53. In response to cellular stress, high levels of SnoN is recruited to the PML nulcear bodies where it induces stabilization of p53, leading to increased cellular senescence and apoptosis [10]. Thus, high levels of SnoN in cells may activate the PML-p53 tumor suppressor pathway to inhibit tumor growth. Finally, SnoN may also inhibit tumor metastasis since reducing SnoN expression enhances epithelial to mesenchymal transition (EMT) of lung and breast cancer cells *in vitro* and tumor metastasis *in vivo* [7].

Given the complex roles of SnoN in tumorigenesis, it is important to determine its expression pattern in normal human tissues and monitor how it changes during malignant progression. SnoN is ubiquitously expressed in all embryonic and adult tissues. Its expression is upregulated during specific stages of embryogenesis and organ morphogenesis [11]. In untransformed cells and normal tissues, SnoN can be detected in both cytoplasm and nucleus, whereas in cancer cell lines, SnoN is largely nuclear [8], [12], [13]. In addition, SnoN expression can be regulated at the level of gene amplification, transcriptional activation and protein stability. The human *snoN* gene is located at chromosome 3q26.2, a locus frequently amplified in many cancer types, including that of esophagus, ovary and many others [5]. In agreement with this, SnoN expression is upregulated in many cancer cell lines [6]. However, whether SnoN expression is also increased in human cancer tissues remains controversial. A number of studies have examined this issue [13]–[18], but no consistent pattern has emerged. While some studies reported an increase in SnoN RNA and protein levels in some cancer tissues and that this higher SnoN expression correlated with poor differentiation, deeper invasion and poor patient survival [13], [14], [16], others detected a decrease in SnoN expression in similar cancers, particularly in dysplastic and highly invasive cancers [15], [17], [18]. The potential relationship between localization of SnoN and malignant status of cancer also varied from study to study. These conflicting data suggest a more complex relationship between SnoN expression and malignant transformation and justify the need for a broader examination of SnoN expression in normal and cancer tissues of different malignant stages.

In this report, we examined SnoN expression in four types of normal human tissues and matching cancer tissues of various clinical stages of malignancy to assess whether alterations of SnoN expression correlate with tumor malignancy and/or status of p53 inactivation. These four types, esophageal, ovarian, breast and pancreatic tissues, were chosen because SnoN has been implicated in the development of these cancers and/or in the normal functions of these tissues. Esophageal and ovarian cancers often involve amplification of the 3q26 amplicon, and the copy number of the *SnoN* gene as well as SnoN transcript levels have been found to increase in these cancers [14], [16], [19], [20]. However the expression pattern of SnoN protein in normal ovary and in ovarian adenocarcinoma have not been defined. Pancreatic cancer is closely associated with the TGF-β/Smad pathway, and Smad4 is inactivated in nearly 60% of pancreatic cancer [21]. As a negative regulator of Smad4, SnoN may also play a role in the development of pancreatic cancer. Finally, our recent studies using various mouse models have shown that SnoN expression is dynamically regulated during mammary gland development [8] and is implicated in the malignant progression of breast cancer [7], [8], [12]. By examining the location and levels of SnoN expression in these tissues, we hope to gain a better understanding of the functions that SnoN play during tumorigenesis in both the epithelium and stromal environment and how this may correlate with p53 inactivation.

## Materials and Methods

### Normal and Cancer Tissue Arrays

All arrays were purchased from Biomax (Cybrdi Inc). These arrays come with information on clinical stages, pathology grades and TNM. The esophageal array contains pairs of tumor biopsy samples and matched normal samples from 39 patients with histologically and clinically classified adenocarcinoma: 8 patients with grade I, 19 patients with grade II, and 11 patients with grade III tumors.

The ovarian tumor array contains tumor biopsy samples and 10 normal matched samples from 69 women aged 50+/−10. The tumors were classified to be ovarian adenocarcinomas: 3 patients with ovarian clear carcinoma, 12 patients with grade I, 29 patients with grade II, and 25 patients with grade III adenocarcinomas (all with lymph node metastasis but not distant metastasis).

The pancreatic tumor array contains samples from 90 patients and five normal pancreatic tissue samples. The tumor types on this array include ductal adenocarcinoma, mucinous adenocarcinoma, neuroendocrine carcinoma, adenosquamous carcinoma, carcinoid, and solid pseudo-papillary carcinoma. They have been classified into different clinical classes based on histological evaluation: 21 patients with grade I, 59 patients with grade II, 8 patients with grade III (many with lymph node metastasis), and 2 patients with grade IV tumor with distant metastasis.

The breast cancer array contains duplicated tumor biopsy samples from 31 patients, with six normal samples from six matching patients, and 9 metastatic samples from 9 matching patients. These tumors were clinically classified by histological analysis to be infiltrating ductal carcinoma: 9 patients with grade IIA, 4 patients with grade IIB, 10 patients with grade IIIA, 5 patients with grade IIIC, 9 matching patients with lymph node metastatic, and 1 patient with metastatic adenocarcinoma to the ovary. Because no difference in SnoN expression was detected between grade IIA and IIB or between grade IIIA and IIIC tumors, we grouped these samples together as grade II or grade III tumors. In addition, 24 pairs of breast ductal carcinoma in situ (DCIS) samples with matching normal tissues were obtained from UCSF cancer center.

The thickness of all the arrays is 5 µm. The breast cancer array contains duplicate samples from each patient and esophageal, ovarian and pancreatic arrays have one section from each patient.

### Immunohistochemistry

#### For SnoN immuostaining

Paraffin-embedded arrays were deparaffinized in xylenes then rehydrated in a series of ethanol gradient. Sections were immersed in 0.85% Nacl for 5 minutes, then in PBS for 5 minutes, and fixed with 4% paraformaldehyde (PFA). Permeabilization of the slides was carried out with 20 µg/ml proteinase K for 10 minutes at room temperature (RT), followed by washing in PBS for 5 minutes. Samples were fixed a second time with 4% PFA, and blocked overnight in 1%BSA/10% newborn calf serum/0.02% Triton X-100 in PBS in a humidified chamber at 4°C. The staining was performed with an anti-SnoN antibody recognizing a C-terminal peptide of SnoN [12] at a concentration of 1 µg/ml for 2 hours along with a peptide competition control and a negative control without primary antibody. After 3 washes with IF buffer (10% newborn calf serum/0.02% Triton X-100 in PBS) for 15 minutes each, fluorescent conjugated secondary antibody (Alex488 anti-rabbit antibody 1∶400 dilution) was added to the slides for 1 hour at RT in the dark. Slides were washed once with IF buffer for 15 minutes and twice with PBS for 10 minutes each, followed by addition of one drop of Vectashield DAPI mounting medium (H-1200; Vector Laboratories, Burlingame, CA).

The sections from the same tissue types (normal and cancerous) were all stained in the same experiment under identical conditions, and images were taken under the confocal microscope in one session. In addition, the same antibody lot and dilutions were used for all stainings described in this paper. Under no circumstances was the antibodies re-used in any of the experiments.

#### For p53 immunohistochemistry

Paraffin-embedded sections were deparaffinized in xylenes, rehydrated in ethanol, and incubated with 3% H_2_O_2_ for 10 minutes to quench endogenous peroxidase activity. Then sections were microwaved at 95°C for 30 minutes in sodium citrate buffer (pH 6) [22] and blocked using the Tyramide Signal Amplification Biotin System Kit (Perkin Elmer, Boston, MA) for 30 minutes at RT. Slides were treated for 30 minutes with anti-p53 at 1∶200 dilution (DO-7 clone; Abcam, Cambridge, UK). After washing 3×5 min in 0.05% TritonX in PBS, slides were incubated in biotinylated secondary antibody for 30 min at RT. All subsequent steps were performed per manufacturer’s protocol. For visualization, DAB was used as the peroxidase substrate (SK-4105; Vector Laboratories, Burlingame, CA).

### Image Capture and Semi-Quantitative Data Analysis

For each SnoN-stained tumor sample, at least three images were captured using the LSM 510 confocal microscope under the 20x objective to make sure that the major areas for each sample are included in the quantification. SnoN intensity (the number of pixels/area) of each of the three areas was calculated using the image J software, and the size of the area was kept constant throughout the quantification process. The numbers were then averaged for each tumor sample. The negative control and peptide competition background signals were substracted from all the samples during quantification. The p53 images are taken with the Zeiss AxioImager fluorescence microscope. Nuclear p53 signal intensity was quantified in a 0 (negative) to 5 (highly intense staining) scale.

For analysis of potential association of TP53 mutation and SKIL amplification, we performed data mining of the Novartis cell line encyclopedia (CLE) that contains 947 human cancer cell lines [23], in which copy numbers and mutations of SnoN (SKIL) and p53 genes have been characterized using Affymetrix SNP6 microarray or RNAseq. We analyzed 914 cell lines by separating the cell lines based on the frequency of SnoN amplification and examining the frequency of TP53 mutation within each group.

Statistical analysis was performed using the student’s t-test function in bioconductor R package as 2-tailed t-tests assuming unequal variances. Pearson’s correlation coefficient was calculated using the R package, and the significance of the observed correlation coefficient was determined using one-sample t-test against the population of correlation coefficients in-between p53 and all Entrez genes in the human genome. Potential correlation between SnoN expression and p53 inactivation in cancer tissues was evaluated using the Kruskal-Wallis test.

## Results

### SnoN is Expressed in Normal Mammalian Tissues

To determine a normal pattern of SnoN in various human tissues and establish a baseline for further analysis of cancer tissues, we first examined SnoN expression in 39 normal esophageal tissue samples, 10 normal breast samples, 6 normal ovarian and 5 normal pancreas samples by immunohistochemistry with anti-SnoN. This affinity-purified antibody was raised against a C-terminal peptide of human SnoN and has been previously characterized for imunofluorescence and immunohistochemistry studies [8], [12].

**Figure 1:**
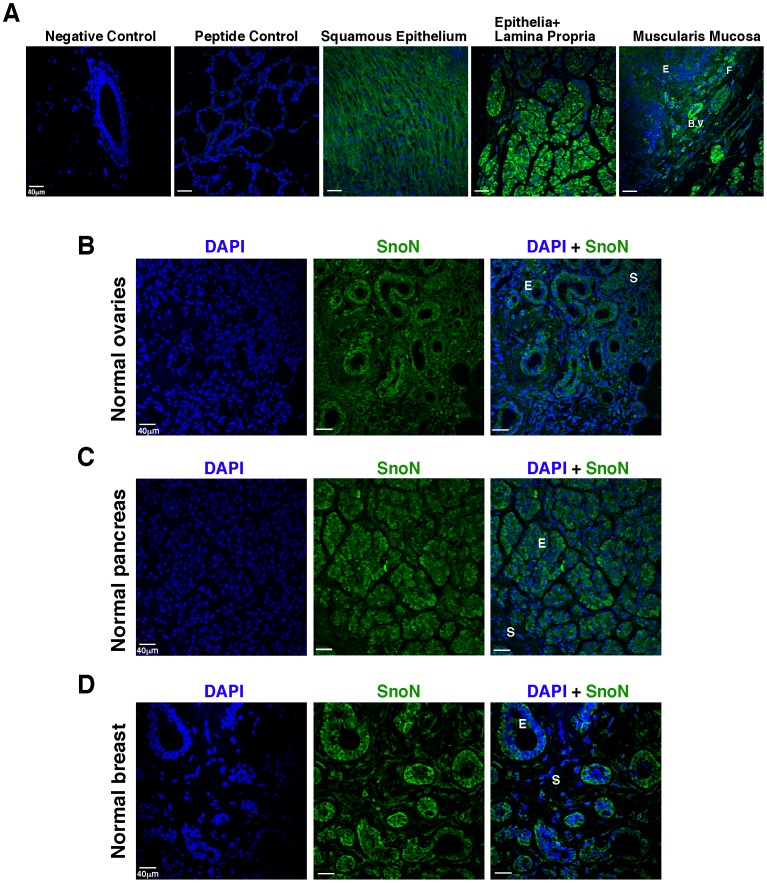
SnoN is expressed in normal mammalian tissues. A, SnoN expression in the normal esophagus, including the suprabasal differentiated squamous epithelial cells, the lamina propria (stroma and connective tissue), and muscularis mucosa (smooth muscle). E: epithelial cells; F: fibroblasts; B.V; blood vessel. Negative control: tissue stained with conjugated secondary antibody alone and without primary antibody. Peptide control: tissue stained with the SnoN peptide competition control. Green: SnoN; blue, DAPI. **B,** Representative SnoN expression in the normal ovarian tissue. E: follicle epithelial cells; S: stroma. The left panel is DAPI stain alone (blue), the middle panel is SnoN stain alone (green), and the right panel is SnoN (green) plus DAPI (blue) stains. Same is true for figure panels in C-D. **C,** Representative SnoN expression in the normal pancreas. E: acinar epithelial cells; S:stromal cells of the lobular connective tissue septa. **D,** Representative SnoN expression in the normal breast. E: epithelial cells of ducts and lobuli; S: stroma.

The mucosa of the **esophagus** contains three layers: the non-keratinized stratified squamous epithelium, the lamina propria consisting of loose connective tissue, and the muscularis mucosa consisting of smooth muscle. In the esophageal stratified squamous epithelium, SnoN was expressed at moderate to high levels in the cytoplasm, with a stronger staining in the upper surface part of the flattened epithelium than in the lower layer of cuboidal cells (Figure 1A). In the lamina propria, SnoN was predominantly localized in the cytoplasm of cells in the connective tissue, but can also be detected in the nucleus of some fibroblasts. Blood vessels expressed SnoN at a high level, and the skeletal muscle of the muscularis mucosa had the highest SnoN staining (Figure 1A). Thus, SnoN is expressed in both epithelial cells and the stromal compartment, and given its high levels of expression in muscles, blood vessels and fibroblasts, it may play an important role in regulating the stromal microenvironment.

In the normal **ovarian** tissue, SnoN was predominantly cytoplasmic and expressed at moderate levels in the primordial and primary epithelial follicle cells and stromal cells (Figure 1B). In the normal **pancreas**, SnoN was present in the cytoplasm of the epithelial cells of the acini and intralobular ducts at moderate levels, and in the lobular connective tissue septa at a lower level (Figure 1C). Finally, in normal **breast** tissues, SnoN was expressed in the cytoplasm of epithelial cells of the lobuli and terminal ducts (Figure 1D), similar to what has been reported before [8], [12]. It was also detected in the stroma at a lower level. Thus, SnoN is present in all the epithelial and stromal layers of these four normal tissue types at varying levels. In the epithelial cells of all tissue types, SnoN is predominantly localized in the cytoplasm. This is consistent with our earlier findings [8], [12].

### SnoN Expression is Reduced in Lower Grade Esophageal Adenocarcinoma but Comparable to that in WT Tissues in the High Grade Tumor Stroma

**Figure 2:**
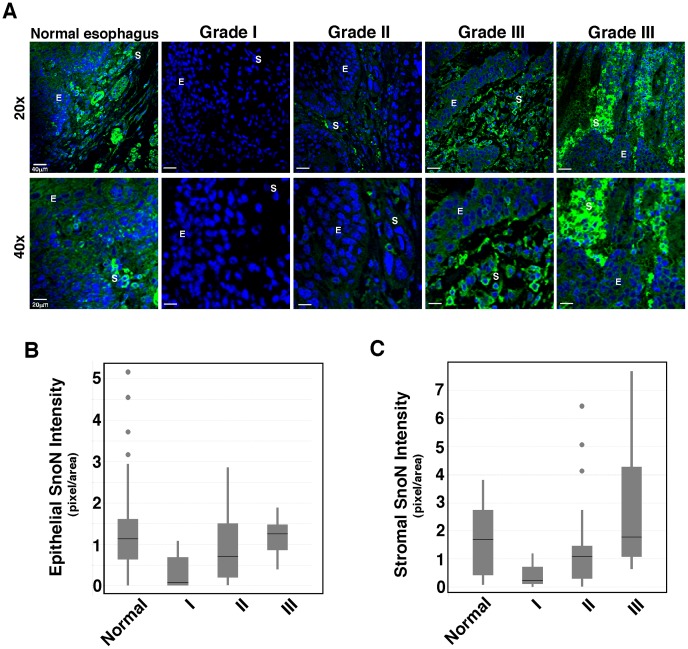
SnoN expression in esophageal adenocarcinoma. **A,** Representative SnoN staining of esophageal cancer of various grades at 20X (top) or 40X (bottom) magnifications. Two grade III samples representing different levels of SnoN expression were shown. E: epithelium; S: stroma. Green: SnoN; blue, DAPI. **B,** SnoN staining in normal and tumor epithelial cells was quantified using the Image J software and the numbers were plotted in the box plot, which includes normal samples (n = 36, mean intensity = 1.13) and esophageal tumor samples of grade I (n = 8, mean = 0.07), II (n = 19, mean = 0.71), and III (n = 11, mean = 1.26). Statistical analysis comparing the normal controls to each tumor grade showed that the epithelial SnoN levels in esophageal adenocarcinoma are significantly weaker (grade I: p = 0.0002) or similar (grade II: p = 0.1425 and grade III: p = 0.3349) to that in the control normal samples. The increase in epithelial SnoN expression in grade III compared to grade I was statistically significant (p = 0.0013)**.** **C,** Quantification of SnoN stromal staining in normal samples (n = 27, mean = 1.69) and esophageal tumor samples of grade I (n = 5, mean = 0.23), II (n = 19, mean = 1.09), and III (n = 11, mean = 1.78). The statistical analysis comparing the normal controls to each esophageal tumor grade is as follow: p = 0.0023 for grade I, p = 0.8565 for II, and p = 0.1132 for grade III. The increase in stromal SnoN expression in grade II (p = 0.0287) and grade III (p = 0.0068) tumors compared to grade I tumor stroma was statistically significant.

We next examined the SnoN expression levels and patterns in 39 esophageal adencarcinoma tissue samples by immunofluorescence staining followed by confocal imaging at both 20X and 40X magnification and compared them with that in the matching normal samples. These esophageal adenocarcinoma samples have been classified clinically from grade I to III. Notably, the multi-layered tissue structure seen in normal esophageal tissue has been lost completely in these adenocarcinoma samples, and tumor stromal cells are interspersed with tumor epithelium. Our semi-quantitative analysis showed that compared to the normal squamous epithelium, SnoN expression was significantly reduced in grade I (p = 0.00016) and to a lesser extent, grade II tumors (p = 0.142) (Figure 2A and 2B). Interestingly, as tumors progressed to a more malignant stage (grade III), SnoN expression recovered gradually to a level comparable to or higher than that in normal controls (two representative samples, one similar to normal and one higher are shown in Figure 2A). SnoN expression in the stromal cells and connective tissue in grade I and II tumors were also decreased significantly from that in normal tissues. Grade III tumor stroma, however, showed a significant upregulation of SnoN expression than grade I tumors (p = 0.0068), reaching a level similar to and in 55% of the samples, higher than that in normal tissues (last panel, Figure 2A), especially in infiltrating fibroblasts and inflammatory cells (lymphocytes, leukocytes and phagocytes) (Figure 2A and 2C). However, due to the large variations among individual grade III samples, the mean change in stroma SnoN levels over that in WT tissues was not statistically significant. Notably, SnoN remained largely cytoplasmic in these tumor samples. Our data suggest that SnoN expression is downregulated in patients with low grade esophageal adenocarcinoma but its expression is re-established in high grade tumors, in particularly in the stroma (for detailed numbers, see Table 1). This suggests that SnoN expression may vary at different stages of malignancy and that SnoN may play different roles at different stages of tumorigenesis.

**Table 1:** Summary of SnoN expression levels (percentages with no expression, low, intermediate, and high) for each grade within different tumor arrays in both the epithelial and stromal compartments.

Epithelial SnoN	Esophageal Cancer	Ovarian Cancer	Pancreatic Cancer	Breast Cancer
Normal				0% No expression
	26% Low	27% Low	0% Low	14% Low
	37% Intermediate	36% Intermediate	25% Intermediate	43% Intermediate
	37% High	36% High	50% High	43% High
Grade I	75% Low	50% Low	23% Low	
	25% Intermediate	17% Intermediate	50% Intermediate	
	0% High	33% High	27% High	
Grade II				67% No expression
	47% Low	30% Low	46% Low	8% Low
	26% Intermediate	40% Intermediate	37% Intermediate	17% Intermediate
	26% High	30% High	18% High	8% High
Grade III				50% No expression
	10% Low	27% Low	38% Low	25% Low
	60% Intermediate	23% Intermediate	25% Intermediate	19% Intermediate
	30% High	50% High	38% High	6% High
Metastatic				60% No expression
				30% Low
				0% Intermediate
				10% High
**Stromal SnoN**	**Esophageal Cancer**	**Ovarian Cancer**	**Pancreatic Cancer**	**Breast Cancer**
Normal				0% No expression
	30% Low	25% Low	50% Low	20% Low
	19% Intermediate	50% Intermediate	50% Intermediate	40% Intermediate
	53% High	50% High	0% High	40% High
Grade I	60% Low	71% Low	20% Low	
	40% Intermediate	0% Intermediate	55% Intermediate	
	0% High	29% High	25% High	
Grade II				62% No expression
	37% Low	38% Low	39% Low	8% Low
	42% Intermediate	42% Intermediate	37% Intermediate	23% Intermediate
	21% High	21% High	24% High	8% High
Grade III				27% No expression
	10% Low	20% Low	50% Low	47% Low
	30% Intermediate	30% Intermediate	50% Intermediate	20% Intermediate
	60% High	50% High	0% High	7% High
Metastatic				60% No expression
				30% Low
				10% Intermediate
				0% High

### SnoN Expression is Reduced in Lower Grade Ovarian Adenocarcinoma Tissues but Elevated in High Grade Tumor Stroma

**Figure 3:**
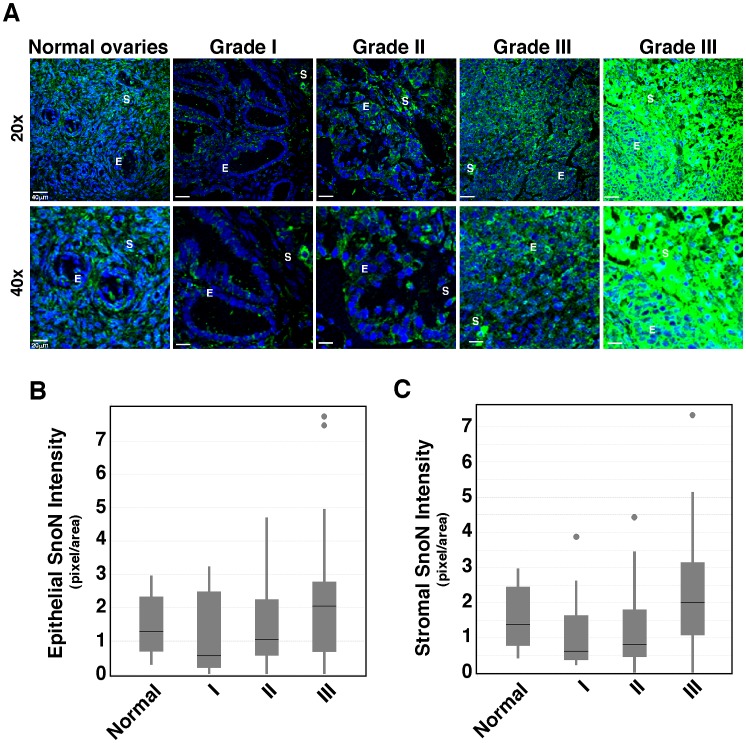
SnoN expression in ovarian adenocarcinoma. **A,** Representative SnoN expression in the normal ovarian tissue and in ovarian adenocarcinoma of various tumor grades at 20X (top) or 40X (bottom) magnifications. Two grade III samples representing different levels of SnoN expression were shown. E: epithelium; S: stroma. Green: SnoN; blue, DAPI. **B,** SnoN staining in normal and ovarian tumor epithelial cells was quantified using Image J software and the numbers were plotted in the box plot, which includes normal samples (n = 10, mean = 1.28) and ovarian tumor samples of grade I (n = 11, mean = 0.55), stage II (n = 29, mean = 1.05), and stage III (n = 25, mean = 2.04). Statistical analysis comparing the normal controls to each tumor grade showed that the epithelial SnoN levels in ovarian adenocarcinoma are equal or weaker than the control tissue samples: p = 0.6139 for grade I, p = 0.7984 for II, and p = 0.1461 for III. No significant difference was observed between the ovarian tumor samples. **C,** SnoN staining in stromal cells in normal samples (n = 10, mean = 1.38) and ovarian tumor samples of grade I (n = 7, mean = 0.61), II (n = 23, mean = 0.81), and III (n = 20, mean = 2.00). The statistical analysis comparing the normal controls to each ovarian tumor grade is as follow: p = 0.5997 for grade I, p = 0.3633 for grade II, and p = 0.1833 for grade III. The increase in stromal SnoN expression in grade III compared to grade II (p = 0.0258) was statistically significant.

Similar to what have been observed in esophageal cancer, SnoN expression was downregulated in grade I and to a lesser degree, grade II ovarian adenocarcinoma samples in both tumor epithelia and stroma (Figure 3 and Table 1), but recovered again in grade III tumors. Grade III ovarian adenocarcinoma samples showed heterogeneity in SnoN expression, with 60% of the samples displaying a higher SnoN staining (last panel, Figure 3A) than the normal tissues and others exhibiting a weaker SnoN staining (panel #4, Figure 3A). As a result of this heterogeneity, statistical analysis showed that there was no overall difference in SnoN expression between normal ovarian tissue and grade III tumor samples. However, when compared to the lower grade tumor samples, the stromal compartment of the grade III tumor samples showed a much stronger SnoN staining (Figure 3A and 3C). These stromal cells were identified by pathologists as infiltrating fibroblasts and inflammatory cells. Again, SnoN remained largely cytoplasmic in these tumor samples. Thus, similar to that has been observed with esophageal cancer, SnoN expression in ovarian cancer also appeared to be lower in early stages of malignant progression.

### SnoN Expression is Decreased in Pancreatic Adenocarcinoma Samples

**Figure 4:**
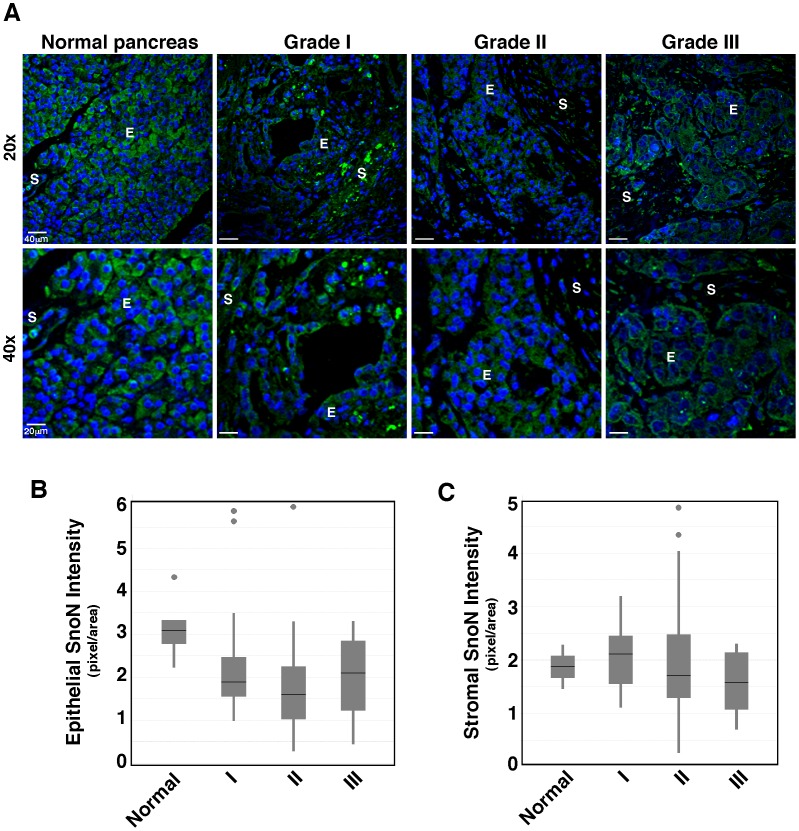
SnoN expression is reduced in pancreatic adenocarcinoma samples. **A,** Representative SnoN expression in the normal pancreas and in pancreatic adenocarcinoma of varying grades at 20X (top) or 40X (bottom) magnifications. E: epithelium; S: stroma. Green: SnoN; blue, DAPI. **B,** SnoN staining in normal and pancreatic tumor epithelial cells was quantified using Image J, and the numbers were plotted in the box plot, which includes normal samples (n = 5, mean = 3.08) and pancreatic tumor samples of grade I (n = 21, mean = 1.89), grade II (n = 59, mean = 1.59), and grade III (n = 8, mean = 2.09). SnoN expression in tumor samples was weaker than that in normal pancreatic samples (p = 0.0855 for grade I, p = 0.0125 for II, and p = 0.0518 for III). No significant difference was observed in SnoN epithelial staining between the pancreatic tumor samples. **C,** SnoN staining in normal (n = 2, mean = 1.87) and tumor stromal samples of grade I (n = 20, mean = 2.10), II (n = 55, mean = 1.70), and III (n = 8, mean = 1.57). There is no statistically significant difference between tumor and normal stroma samples.

The pancreatic cancer tissue array contains multiple types of adenocarcinoma of all three clinical grades (I-III), including ductal adenocalarcinoma, mucinous adenocarcinoma, neuroendocrine carcinoma, adenosquamous carcinoma, carcinoid, and solid pseudo-papillary carcinoma. In all pancreatic cancer samples across all three tumor grades, SnoN was cytoplasmic and was expressed at a lower level than that in the normal age-matched samples (Figure 4A–4B and Table 1). Across the tumor grades, SnoN expression was the highest in stage III pancreatic adenocarcinoma (Figure 4B). However this increase was not above the normal tissue controls. The tumor stromal compartment did not have a higher SnoN staining than the normal stroma (Figure 4A and 4C). Thus in none of the tumor tissues was SnoN expressed at a higher level than that in normal tissues, consistent with a tumor suppressor role of SnoN in pancreatic cancer.

### SnoN Expression is Significantly Reduced in Breast Ductal Adenocarcinoma

**Figure 5:**
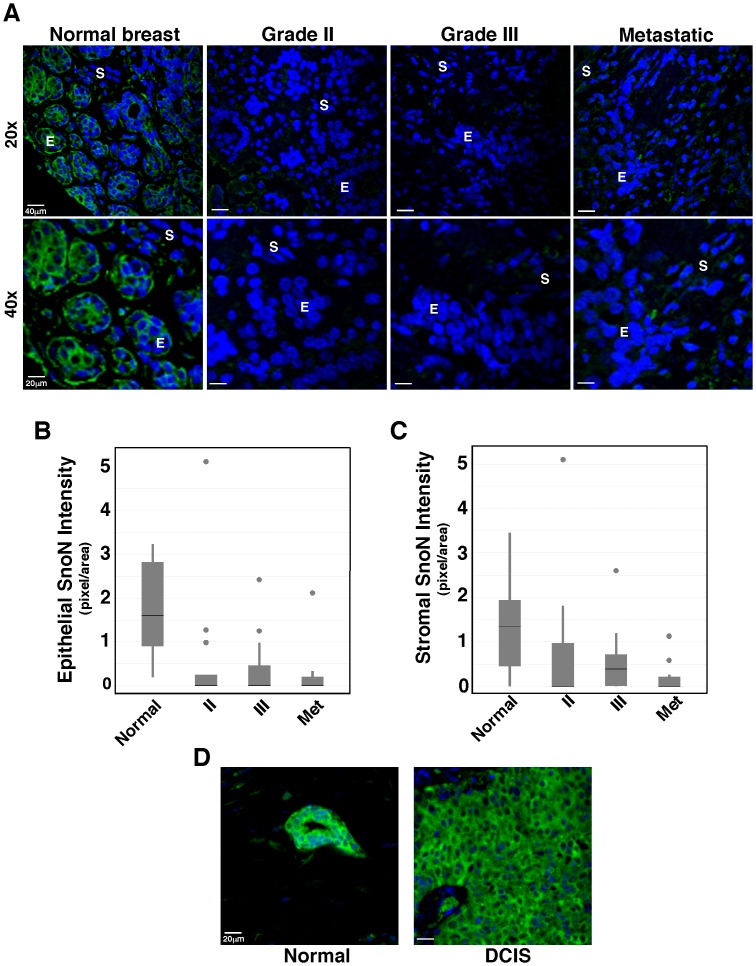
SnoN expression is reduced in breast ductal adenocarcinoma. **A,** Representative SnoN expression in the normal and breast ductal adenocarcinoma samples at 20X (top) or 40X (bottom) magnifications. E: epithelium; S: stroma. Green: SnoN; blue, DAPI. **B,** SnoN staining in normal (n = 6, mean = 1.59) and breast tumor epithelial cells of grade II (n = 9 for IIA and n = 3 for IIB, mean = 0), grade III (n = 10 for IIIA and n = 5 for IIIC, mean = 0), and metastatic samples (n = 10, mean = 0). The decreases of SnoN expression in tumor samples in comparison to normal tissues has a p value of 0.0966 for grade II, 0.0430 for grade III, and 0.0321 for the metastatic samples. **C,** SnoN staining in stromal cells from normal (n = 6, mean = 1.35) and breast tumor samples of grade II (n = 9 for IIA and n = 3 for IIB, mean = 0), grade III (n = 10 for IIIA and n = 5 for IIIC, mean = 0.38), and metastatic samples (n = 10, mean = 0). The p values for each tumor/normal comparison was 0.3340 for grade II, 0.1647 for grade III, and 0.0695 for the metastatic samples. **D,** Representative SnoN expression from a normal breast section and DCIS section taken from the same patient. Green: SnoN; blue, DAPI.

The breast cancer tissue array contains duplicated biopsy samples from patients with infiltrating breast ductal carcinoma of stages II and III and metastatic carcinoma to the lymph nodes. To our surprise, SnoN levels were not elevated in any of the tumor samples (Figure 5A–5B and Table 1). On the contrary, SnoN levels were significantly lower than the control samples and no correlation was seen between its expression levels and the presence of lymph node metastases (Figure 5B). SnoN was expressed at high levels in very few samples and was predominantly cytoplasmic. For most of the tumor samples, SnoN expression in the stroma was markedly lower than that in the normal control samples (Figure 5A and 5C).

To test the possibility that SnoN may be elevated during early stages of tumorigenesis but reduced at late stage of malignant progression, we collected and stained 24 human DCIS samples and their matching normal tissues for SnoN expression. Interestingly, although in any individual cell in the DCIS tissues, the level of SnoN expression was similar to or in a few cases, lower than that in cells in normal tissues, due to a markedly increased epithelial content in DCIS samples, the overall SnoN level in the whole tissue was much higher than that in normal tissues (Figure 5D). Thus, the overall increase in SnoN level in DCIS samples reflects the significantly elevated epithelial contents in these tissues but not the upregulation of SnoN expression in individual cells.

Taken together, unlike previously proposed, SnoN expression is reduced in lower grade malignant tumors of esophagus, breast, pancreas and ovary (Table 1). In esophageal and ovarian cancer, and to a lesser degree in pancreatic cancer, SnoN expression gradually recovered in grade III tumors. This finding is consistent with the model that SnoN may function initially as a tumor suppressor in malignant progression, possibly through activating p53. The model further predicts that in order for tumor cells to overcome the tumor suppressive activity of the SnoN-p53 pathway, they have to either inactivate p53 or delete SnoN.

### Elevated SnoN Expression Correlates with Inactivation of p53 in Human Cancer Cell Lines but not in Primary Tumor Tissues

**Figure 6:**
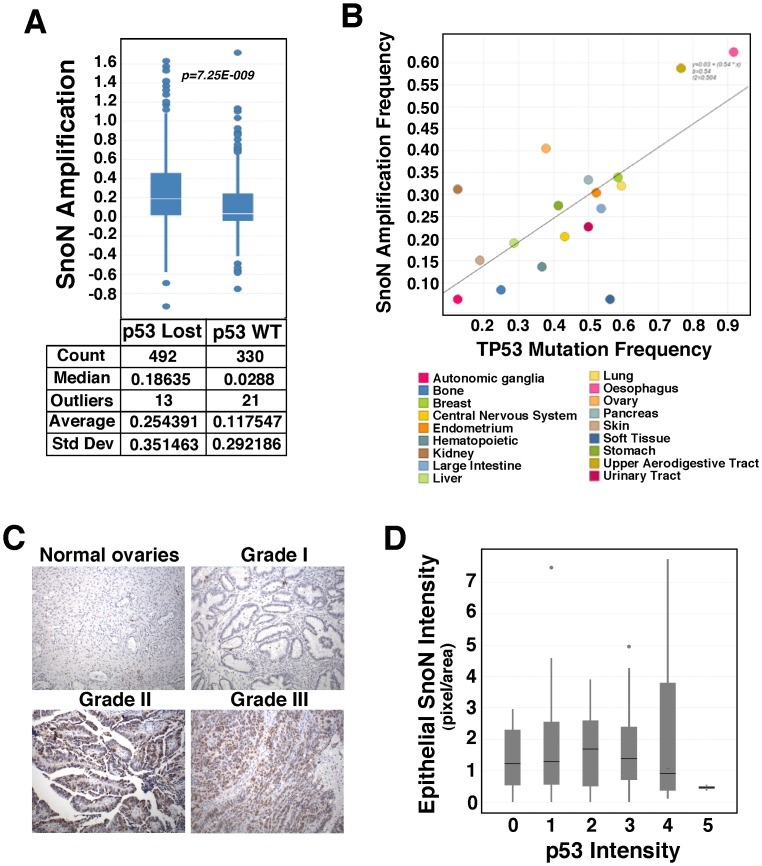
Elevated SnoN expression correlates with inactivation of p53 in human cancer cell lines but not in primary tumor tissues. **A,** 914 cancer cell lines from the Novartis CLE were classified based on their p53 gene status (lost or wild-type) as shown in the X-axis and their correlation with the copy numbers of SnoN as indicated in the Y-axis. A significant enrichment of SnoN amplification events in p53 mutant or deleted cell lines was identified (p = 7.25E-009). **B,** Cell lines from the CLE were divided into 18 different tissue lineages as depicted by various colors, and the correlation between the frequency of TP53 mutation (X-axis) and frequency of SnoN amplification (Y-axis) was determined to be highly significant with a Pearson’s correlation coefficient of 0.7. **C,** Representative p53 immunohistochemical stain in normal ovarian tissue and ovarian adenocarcinoma of grade I, grade II, and grade III (Original magnification ×20). **D,** Box plot depicting the intensity of epithelial SnoN expression (Y-axis) and p53 protein levels (as marked from 0 to 5, 0 being the lowest level in normal tissues and 5 being the highest). No significant correlation between the status of SnoN protein level and p53 inactivation was noted as measured by the Kruskal-Wallis test (p = 0.817).

In contrast to its reduced expression in primary tumor samples, SnoN have been shown previously to be elevated in esophageal, ovarian and breast cancer cell lines [7], [16], [19], [20], due possibly to gene amplification and/or transcriptional activation. Since this elevated SnoN can activate p53 to trigger senescence, these cancer cells are predicted to inactivate this p53-dependent pathway in order to sustain proliferation. We therefore tested the hypothesis that cancer cells with elevated SnoN may also show inactivation of p53. We analyzed 914 cancer cell lines in the Novartis cell line encyclopedia (CLE) [23], in which copy numbers and mutations of SnoN and p53 genes were characterized using Affymetrix SNP6 microarray or RNAseq technology, to determine whether samples with increased copy number of the *SnoN* gene also tend to show inactivation of p53 as indicated by a loss of p53 copy number or the presence of known inactivating mutations. Based on the mutation status of p53, we classified cell lines into mutant or wild type, and copy number of SnoN were compared among these two groups. As shown in Figure 6A, we identified significant enrichment of SnoN amplification events in p53 mutant or p53 deleted cell lines (p value: 7.25E-009), indicating a potential association between the two genetic events. To further assess the association of p53 mutation and SnoN amplification, we partitioned cell lines by lineage into 18 sub-groups, and determined the frequency of SnoN amplification and the frequency of p53 mutation within each group and for all groups. A striking correlation between the frequency of p53 mutation and the frequency of SnoN amplification was detected (The Pearson’s correlation coefficient was 0.7), where the lineages with higher incidence of p53 mutation tended to have higher incidence of SnoN amplification (Figure 6B). Thus, at least in human cancer cell lines, increased SnoN expression correlates with inactivation of p53.

Next we asked whether there was any correlation between SnoN expression and the status of p53 in the four types of human cancer tissue samples that showed reduced SnoN expression by staining the cancer tissue arrays with anti-p53. In all normal tissue samples, p53 was absent as expected (data not shown). In contrast, majority of ovarian tumor samples exhibited a strong p53 nuclear staining, indicating the presence of inactivating mutations [24]. The levels of p53 correlated with the malignant stage of the tumors, but showed no significant correlation between SnoN and p53 expression levels (p = 0.817) (Figure 6C). Similarly, no significant correlation was detected between SnoN expression levels and p53 mutation status in esophageal, breast and pancreatic cancer samples (Figure 6D). This is not entirely surprising as most cancer tissues we tested seem to downregulate SnoN protein. This downregulation of SnoN protein is sufficient to abolish its tumor suppressor activity and therefore presents no need to additionally inactivate the downstream p53.

Together our study suggests that SnoN is likely to play a tumor suppressor role in the initial stages of human cancer development, and inactivation of this pathway by targeting either SnoN itself or p53 is necessary for malignant progression.

## Discussion

In this report, we found that SnoN is expressed in the normal human esophageal, ovarian, pancreatic and breast tissues predominantly in the cytoplasm of both the epithelium and stroma. The intracellular localization of SnoN did not alter significantly in tumor samples, but its expression is downregulated in all four types of tumor epithelia at lower malignant grades, but gradually recovered in higher grade tumors and markedly upregulated in the stroma of esophageal and ovarian cancer tissues. These results suggest that SnoN expression may vary at different stages of tumor progression and that SnoN may play different roles at different stages of malignancy. In particular, the initial downregulation of SnoN in the tumor epithelium is consistent with its anti-tumorigenic activity. The later upregulation of SnoN in the stroma microenvironment in esophageal and ovarian cancers suggests that it may play a role in promoting tumorigenesis in an indirect manner. Thus, the pro-oncogenic activity of SnoN in human cancer may be non-tumor cell autonomous and through affecting the tumor microenvironment.

Our results differ somewhat from those published previously, in particularly those that reported an increase in SnoN levels in cancer tissues [13], [16]. Although the reason for this difference is not known at this time, several possible causes could be speculated. First, the difference could potentially be due to the variations among different samples. Indeed we found that even normal breast tissue samples from different individuals showed a certain level of heterogeneity in term of SnoN levels, possibly due to the different conditions (tissue damage, stress, etc) or treatment procedures that the tissues have experienced. It is therefore quite possible that some cancer samples may also show variations in SnoN levels as a result of the diverse and complex tissue environment under which the tumors develop. However, the overall trend in all four cancer types with over hundreds of tumor samples in our hands is a consistent reduction of SnoN in the tumor epithelium. The level of SnoN may also change depending on the malignant stages of tumors and vary from tissue to tissue. While in esophageal and ovarian cancer, SnoN is downregulated in low grades adenocarcinoma but again elevated in grade III tumors, grade III breast cancer did not show any elevation even when compared to low grade tumors. Similarly, while high SnoN expression is found in stroma of high grade esophageal and ovarian cancer, pancreatic and breast cancer did not show a strong elevation in stroma SnoN expression. Thus, SnoN may play different roles in the progression of different cancer types and possibly through different mechanisms. Another reason may be related to the methods used to measure SnoN expression. Since SnoN expression is upreguated in the stroma while decreased in the tumor epithelium, it is possible that by measuring overall SnoN levels by western blotting, one might detect an increase in the overall expression of SnoN. Finally, the difference could be due to the different antibodies used in the staining. The commercial anti-SnoN antibodies commonly used before showed a high background and sometimes detected nonspecific signals in our hands (data not shown). The anti-SnoN C-terminal peptide antibody has been carefully characterized, and antigen competition experiments demonstrated the specificity for the SnoN signal (Figure 1A) [8], [12]. Moreover, using this antibody, we have detected dynamic changes in SnoN expression during normal tissue morphogenesis [8], [12] and malignant progression, providing additional support that the signals we detect are specific.

Our study has revealed a previously unappreciated role of SnoN in the tumor microenvironment. Especially in esophageal and ovarian tissues, multiple stromal cell types showed high levels of SnoN expression, including smooth muscle cells, blood cells, fibroblasts and infiltrating lymphocytes. Interestingly, the cancers of esophagus and ovary tend to harbor 3q26 amplification and show SnoN copy number increases, and they also display a stronger increase in stroma SnoN levels. Thus, the high SnoN expression in the stroma may be related to gene amplification, and this in turn may enhance malignant progression in these tissues. More and more studies have indicated that tumor-associated stromal cells and infiltrating lymphocytes promote epithelial tumorigenesis, invasion and metastasis, and cancer inflammation [25]–[27]. In addition, infiltrating B- and T-cell lymphocytes can regulate solid tumor development through their abilities to regulate myeloid cell function in solid tumors [28]. Interestingly, TGF-β is a well-known regulator of the tumor microenvironment by modulating the proliferation of fibroblasts, secretion of chemokines and cyokines that control inflammation, recruitment of bone-marrow-derived myeloid cells and host immunosurveillance [29]–[31]. As a negative regulator of TGF-β signaling, it is conceivable that SnoN may also modulate the tumor microenvironment and tumor-host interaction. Our results clearly indicate that this is an important area to focus on in future studies.

High levels of SnoN have been shown to directly stabilize and activate p53. Thus, tissues or cells that show elevated SnoN expression (either due to gene amplification or other means) may cease proliferation due to p53-mediated senescence or cell cycle arrest. Malignant cells have to overcome this barrier in order to maintain their survival and expansion and may do so by either inactivating/deleting SnoN itself or any downstream steps in the SnoN-p53 pathway. Indeed, our analysis of CLE cell lines has indicated a significant correlation between increased SnoN copy number and p53 inactivation, supporting the idea that the tumor suppressive SnoN-p53 pathway must be inactivated to allow malignant progression. A previous study of human breast cancer tissue samples also note a correlation between SnoN upregulation and elevated p53 expression, which is indicative of inativating mutation of p53 [13]. However, in human cancer tissue samples that we have tested, no correlation between SnoN expression and p53 status can be detected. This is not entirely surprising given that these tumor tissues have already downregulated SnoN expression and therefore had no pressing need for additionally inactivating p53.

In summary, we have found that SnoN is expressed in both epithelial and stromal cells in normal human esophageal, ovarian, pancreatic and breast tissues. Unlike what has been depicted in the current models, SnoN expression is downregulated at the protein level in the tumor epithelium in low grade tumors in all four tissues, consistent with its anti-tumorigenic activity. In contrast, SnoN expression is markedly upregulated in tumor stroma in some cancer types, especially at late stages of malignancy. Giving the importance of tumor stroma in influencing malignant progression, this result promopted us to speculate that SnoN may also regulate tumorigenesis through modulating tumor microenvironment.
